# Drug outcomes research and policies – trends and challenges

**DOI:** 10.3389/fphar.2024.1476849

**Published:** 2024-08-26

**Authors:** Bernd Rosenkranz

**Affiliations:** ^1^ Fundisa African Academy of Medicines Development, Cape Town, South Africa; ^2^ Institute for Clinical Pharmacology and Toxicology, Charité Universitätsmedizin, Berlin, Germany; ^3^ Division of Clinical Pharmacology, Faculty of Medicine and Health Sciences, Stellenbosch University, Cape Town, South Africa

**Keywords:** outcomes research, pharmaceutical policies, effectiveness trials, health technology assessment, patient-reported outcomes, healthcare delivery, equity

Outcomes research quantifies the results of medical treatments and/or interventions and their benefits and risks in patient populations to optimize patient outcomes and healthcare delivery and to assist all stakeholders (clinicians, patients, healthcare providers and managers, funders, policymakers and government) to make informed decisions. Outcomes research can be economic (pharmacoeconomics), clinical (comparative clinical effectiveness research), or humanistic (health-related quality of life) (Krumholz et al., 2005; Academy of Managed Care Pharmacy, 2024). It provides real-world data to support the concepts of fairness and equity (Universal Health Coverage, UHC), evidence-based healthcare, personalised medicine, and health technology assessments/cost-effectiveness. By addressing individual patient care and global health, outcomes research is aligned with the United Nations Sustainable Development Goals (SDGs) adopted in 2015, most importantly with SDG 3 (Good Health and Wellbeing), but also with societal and economic ones (SDG 5 (Gender equality), SDG 9 (Industry, Innovation and Infrastructure), SDG 10 (Reduced Inequalities), and SDG 12 (Responsible Consumption and Production). This Editorial focusses on current trends and challenges in outcomes research and policy development.

Medicines development programmes are designed to demonstrate quality, safety and efficacy of a novel medicinal product to obtain marketing authorisation approval. Traditionally, at least two adequate and well controlled efficacy trials in selected patient populations under strictly controlled conditions are required (Clancy and Eisenberg, 1998; Naci and Forrest, 2023). However, what is considered adequate for regulatory approval does not provide all information necessary for treatment of a diversity of patients in different regions in the world. Research efforts after marketing authorisation assess safety and effectiveness of medicines as they are disseminated beyond the clinical trial environment. In this way, phase 3 clinical efficacy trials are complemented by outcomes research to develop health policies that are safe, effective, patient-focused and cost-effective, and will often lead to re-evaluation of prior evidence and adjustments in practice, treatment guidelines and policy (Krumholz, 2009).

Although the understanding of „outcomes research” has evolved over time and there is no unambiguous definition of this term (Jefford et al., 2003), there is consensus that it is defined by its aims and objectives, effectiveness of public-health interventions (including medicines) and health services, related to the individual patient perspective or to effectiveness and economic considerations of healthcare delivery (Kolte, 2017). The appropriate research methodologies to achieve these goals are applied, such as observational studies, randomized clinical trials, cost-effectiveness measures, meta-analysis, pharmacometrics and modelling, surveys, patient registries, assessment of health status or burden of disease studies. Clinical treatment outcomes are assessed in drug effectiveness trials, which - in contrast to trials designed for marketing approval - test treatments across a spectrum of patients in real-world conditions with follow-up periods that match typical treatment regimens, and therefore provide critical information on drug effects in those patients who may ultimately receive the treatment. In these studies, the choice of an appropriate local comparator treatment (“gold standard”) is critical.

Outcomes research forms an essential part of health technology assessments (HTA) to evaluate whether the product works better, equally well, or worse than existing alternatives. Outcomes include therapeutic and adverse effects, quality of life and patient-reported outcomes (Kastien-Hilka et al., 2016), but also cost implications and impact on the healthcare systems. This information is used by national authorities and private sector payors to decide which medicines or technologies should be reimbursed at national level (https://health.ec.europa.eu/health-technology-assessment/overview_en).

WHO recommend that all countries formulate and implement a comprehensive national medicines policy as a means to improve access to safe, effective medicines of good quality (https://www.who.int/teams/health-product-policy-and-standards/medicines-selection-ip-and-affordability/medicines-policy). The role of pharmaceutical policies is to ensure reliable and consistent availability of medicines, appropriateness of prescriptions, affordability of drugs and protection of patients against catastrophic expenditure (Gautier and David, 2022). In an Editorial accompanying the Frontiers in Pharmacology Research Topic “Pharmaceutical Policy, Impact and Health Outcomes”, Kwon and Godman (2023) discuss the role of pharmaceutical policy in addressing increasing expenditure on medicines and only finite resources. The case studies published in this Research Topic demonstrate the interest across countries to develop and implement pharmaceutical policies to enhance the rational use of medicines.

What are the greatest opportunities and challenges in outcomes research and policy development? As described in a recent editorial by the editors-in-chief of four scientific journals (Abraham et al., 2023), current trends in health economics and outcomes research are centered around alternative and novel and fair metrics of benefit and harm (example: improvement in severely ill vs. moderately ill patients), patient preferences and adherence, real-world evidence, access and equity to therapeutics in low- and middle income countries, economics of prevention, and evaluating and assessing medications for rare conditions.

Major challenges remain the assessment of real-world treatment outcomes in vulnerable patient groups (such as children or elderly) or communities (such as ethnic minorities), and regional socio-economic or cultural differences, especially in low-, middle-income or developing countries. The magnitude, extent, and distribution of these factors associated with health outcomes have often not been tested in diverse populations, and more research will be necessary to assess these.

Innovative analysis methods include pharmacometrics and modelling techniques (e.g., time course of diseases, real-world treatment patterns) (Frontiers in Pharmacology Research Topic „Pharmacometrics–Tools to assure optimal medicine use in low- and middle income countries”; Abulfathi et al., 2022). These techniques provide an opportunity for further in-depth evaluation of treatment outcomes, but require adequate validation and sensitivity analysis (Laurent et al., 2023). The use of accessible databases (big data research) is hampered by concerns related to their quality and completeness.

Another novel challenge is the use of Artificial Intelligence (AI) in outcomes research for planning and interpretation of additional studies which will improve the evidence base and advance health equity and access to treatments (Graili et al., 2021). AI solutions are developed for outcomes research across therapeutic areas. These should be aligned with fair, appropriate, valid, effective, and safe (FAVES) AI principles as outlined by the US Department of Health and Human Services (US DHHS, 2023).

In a Frontiers in Pharmacology Opinion Piece, Sarri (2022) described health inequalities demonstrated by population or community real-world evidence studies for all countries around the globe to a varying degree. Inequalities are related to unaffordability of therapeutic interventions and poor health literacy, but also the heavy disease burden disproportionally experienced among the less priviledged in the society. A major challenge lies in the planning, funding and implementation of further patient-centered outcomes research initiatives to demonstrate, understand and address inequalities in healthcare delivery and to overcome barriers in policy and decision making by government, funders, researchers, and industry to create a more equitable healthy society.

The recent Covid-19 pandemic as well as other epidemic outbreaks have demonstrated the need for outcomes research, health technology assessments and policy development which is adaptable to public health emergencies.

The development of pharmaceutical policies is complicated by a number of challenges (discussed by Gautier and David, 2022). Examples are the management of uncertainties, the bridging between the diverse clinical, economic and societal outcomes and requirements, the interaction with pharmaceutical industry and questioning of presented research outcomes, the interactions with regulatory agencies and with international stakeholders (such as WHO or WTO, especially for LMICs), as well as political considerations and power relations. Various characteristics of the product value need to be considered, such as perceived vs. actual value for the patient and healthcare systems, economic value of pharmaceuticals mainly regarded as commodities, or social and moral value of pharmaceuticals mainly regarded as public goods. Policy development must take into account both the needs of organizational characteristics of health systems as well as individual clinical patient care.

Heterogeneity in value assessment systems by health technology agencies in different countries resulting in different coverage recommendations must be addressed (Carvalho et al., 2021). Engagement of patients and patients advocacies and collaboration between academic researchers, industry partners, and regulatory agencies are strongly encouraged (Pizevska et al., 2022; Piemonti et al., 2023).

It is important that the practical implementation of health policies and governance concerns are considered in the analyses. If this is not addressed, the policy suggestions may be financially sensible, but politically unrealistic or socially unsuitable for the intended population. This has been especially a matter of concern in LMICs (Al Meslamani, 2024).

The growing number of advanced therapy medicinal products (ATMPs) provide new challenges for outcomes research and policy development. ATMPs for human use include cell and gene therapy products (CGTPs), such as the novel gene editing products, and tissue engineered products that are substantially manipulated and/or perform different functions in the recipient than in the donor (WHO, 2023). Often, only limited data are available for these products, because of the low number of patients or the fact that long-lasting effects require very long follow-up for many years. The scarcity of clinical information can be an obstacle in patient access (Carvalho et al., 2021). Adequate patient follow-up by post-authorisation real-world evidence studies is mandatory for continued benefit-risk assessment and to support reimbursement (Pizevska et al., 2022), an important patient access hurdle.

Improvement of patient access to ATMPs and CGTPs can only be achieved by understanding all hurdles (Carvalho et al., 2021), by changes at the policy level, and by health economic innovation. The dichotomy of quality of care and cost containment is particularly challenging for CGTPs. Examples are the once-off gene treatments Zolgensma™ (onasemnogene abeparvovec-xioi) approved for treating spinal muscular atrophy in 2020 at $2.1 million (Rigter et al., 2021), and Lenmeldy™ (atidarsagene autotemcel) approved for treating metachromatic leukodystrophy (MLD) in 2024, currently at $4.25 million the most expensive drug worldwide (Bansal, 2024). Gene therapies for rare conditions and other extremely costly novel therapies can transform health for the better, but this requires rapid adaptation of health systems, pharmaceutical research and development financing, and reimbursement policies to address the cost challenges (Pearson, 2024). Special solutions will be necessary for LMICs.

Efforts to harmonise development, application and the regulatory framework of ATMPs, including CGTPs have recently been initiated by WHO (WHO, 2023) and the European Cooperation in Science and Technology (COST) Action (“Genome Editing to treat Human Diseases“, GenE-HumDi network) (Cavazza et al., 2023).

A repository of health economics and outcomes research (HEOR) resources, such as Good Practice guidelines, is available from The Professional Society for Health Economics and Outcomes Research (ISPOR) (https://www.ispor.org/heor-resources).

Outcomes research must balance research needs with privacy and equity and justice concerns. This is particularly relevant for the use of sensitive healthcare data, such as electronic medical records, or for the analysis of healthcare financing, policy for vulnerable populations, and equitable access to care (Al Meslamani, 2024). Strict ethical principles, such as patient confidentiality and transparency must be enforced in clinical outcomes research.

Effective capacity strengthening in outcomes research, medicines regulatory science and policy development, especially in LMICs, is a major challenge (ESSENCE on Health Research and CCR, 2023). This is addressed by a number of initiatives by Universities or other organisations (for example Kerpel-Fronius et al., 2015; Semete-Makokotlela et al., 2021; Najjemba et al., 2023; Pillai et al., 2023; Silva et al., 2024; Mollet et al., 2024), but will require further efforts.

Research outcomes must be communicated to healthcare professionals and to the public in an adequate manner (Abraham et al., 2023). Frontiers in Pharmacology - Drugs Outcomes Research and Policies is a worldwide, open access platform for bioscientific, clinical and sociocultural research. It provides a forum to communicate outcomes research and policies to the scientific community, with a focus on the evaluation of drugs, vaccines, advanced therapies and medical devices containing drugs in real-life conditions, following their approval by regulatory authorities and access to the market. Scientists are very welcome to submit research topics for publication in this section dedicated to the themes described above or any related topics.
